# Analgesic Effect of Carboxytherapy for Postoperative Neuropathic Facial Pain: A Case Report

**DOI:** 10.7759/cureus.26301

**Published:** 2022-06-24

**Authors:** Clara Lopes Machado, Marina Lopes Machado, Lúcia Lourenço Lopes

**Affiliations:** 1 Internal Medicine, Independent Researcher, Vitória, BRA; 2 Dermatology, Independent Researcher, Vitória, BRA

**Keywords:** pain alternative therapies, interventional pain medicine, postoperative pain, carbon dioxide injection, oro-facial pain, chronic postoperative pain, carboxytherapy

## Abstract

Postoperative pain is a major concern in surgical patients and is often challenging to treat. Studies have shown that carboxytherapy may be helpful in some cases of persistent pain, as it increases tissue oxygenation. This report describes the case of a patient who received carboxytherapy after three years of persistent postoperative neuropathic facial pain and successfully had her symptoms reduced.

## Introduction

Approximately 80% of patients who undergo oral and maxillofacial surgery report moderate or severe pain postoperatively [1]. Many strategies have been adopted to manage postoperative pain in these patients, including nerve blockage, nonsteroidal anti-inflammatory drugs (NSAIDs), opioids, local anesthetics, relaxation techniques, and acupuncture [1]. Even with these many therapies available, postoperative pain is still a challenge in this type of surgery, as it can last long periods and be difficult to treat, especially when it comes to neuropathic pain [2].

Transcutaneous application of gaseous carbon dioxide (CO_2_) has been considered for pain management in a few settings [3]. The rationale behind its analgesic properties is that it seems to increase the oxygenation of tissues due to a vasodilatory effect after the CO_2_ application. Besides, it is also believed that the increased carbon dioxide in the area induces hemoglobin to release oxygen through the Bohr effect, contributing to this increased oxygenation of tissues [4].

In this study, we describe the case of a patient with refractory postoperative neuropathic perioral pain after orthognathic surgery in which carboxytherapy was used as treatment and successfully reduced the patient’s symptoms.

## Case presentation

**Figure 1:**
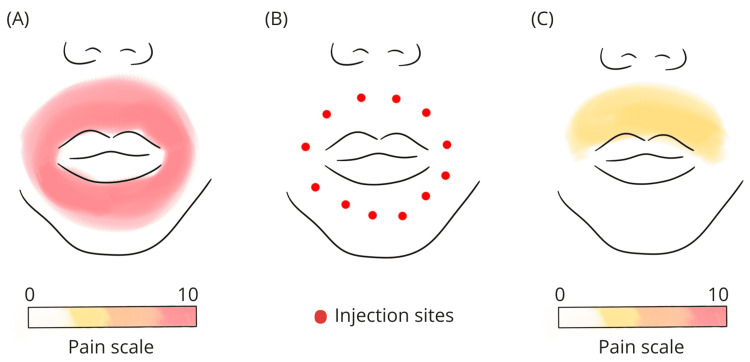
(A) Illustration of pain reported by the patient before carbon dioxide injection. (B) Injection sites of carbon dioxide. (C) Illustration of pain reported by the patient after carbon dioxide injection.

A 67-year-old female presented to our care for regular dermatological treatment. During her appointment, she complained of persistent perioral pain for three years (Figure 1A), which started after she underwent two orthognathic surgeries to realign her jaw and teeth. She stated that the pain started immediately after her second surgery and that she initially attributed it to local swelling. However, the pain persisted after the swelling subsided and the patient described it as constant throbbing pain, intermittent shooting pain, and allodynia, which were suggestive of neuropathic pain. The pain was rated 8 on a Numerical Rating Scale (NRS), where zero represents “no pain” and 10 represents "worst pain possible.” Stress, agitation, and warm temperatures were identified as aggravating factors. A relieving factor identified was the use of moisturizers, but the positive effect quickly vanished after they were absorbed. Many over-the-counter analgesics had been attempted during the three years following the surgery, including acetaminophen (750 mg orally) and NSAIDs (nimesulide 100 mg orally), but the improvement was only partial and temporary. The patient went to a neurologist for evaluation of neuropathic pain and physiotherapy was proposed but the patient did not comply with the therapy.

An experimental session of carboxytherapy in the painful area was proposed to the patient. The technique consisted of 12 intradermal injections of carbon dioxide to cover the entire perioral area (Figure 1B). Each injection lasted approximately three seconds and the flow of CO_2_ used was 150 mL/min. The needle used had a length of 13 mm and diameter of 0.3 mm, and the angle of inclination of the needle during injection was between 10^o^ and 15^o^. After only one session of carboxytherapy, the patient reported complete resolution of pain in the inferior perioral area (NRS: 0) and markedly decreased pain in the superior perioral area (NRS: 4) within one week of the treatment (Figure 1C). The only reported side effect was moderate pain during the procedure.

The patient returned for a follow-up visit two months after the treatment and reported that the analgesic effect remained unchanged since the first week after the procedure.

## Discussion

Postoperative neuropathic pain can be challenging to treat, as it can become chronic and disabling [2]. Adequate treatment is important since severe pain after surgery increases the risk of chronic postoperative pain due to continuous nociceptive stimuli causing neuroplastic changes [1]. Several treatment strategies have been adopted, including pharmacological and psychological therapies, but their results are not always satisfying [2]. Therefore, the development of more effective strategies to control postoperative pain could help improve patients’ quality of life.

Carboxytherapy is a well-known technique in dermatology, and it has been used for several years for both aesthetic and pathological conditions [3-6]. It is considered a simple and safe procedure, with mostly mild and temporary adverse events such as pain and bruising [5]. The use of this technique for the treatment of conditions associated with pain, such as myofascial syndrome and fibromyalgia, has also been described [3], although we found no clinical trials on the subject. A clinical trial that studied the effect of carbon dioxide application in diabetic peripheral neuropathy found that many patients reported improvement in pain although the measured outcomes did not include pain [4]. Therefore, new clinical trials focused on carboxytherapy in the management of pain could help determine its clinical significance.

In this patient, a significant improvement in postoperative neuropathic facial pain was seen with one session of carboxytherapy. However, limitations of this study include the exclusive use of NRS to measure pain improvement, while other factors like sleep and quality of life were not assessed. Furthermore, the patient did not undergo other treatments frequently used in the management of neuropathic pain such as psychological therapies and pharmacotherapy with antidepressants or antiepileptics [2].

Further studies are needed to determine the significance of the analgesic effect of carboxytherapy in larger populations and to study whether or not additional sessions of the procedure could provide added benefit. There is also the need to determine which types of pain could benefit from the treatment and for how long the effect might last.

## Conclusions

Carboxytherapy is a simple and safe procedure that could be promising for pain treatment in some settings. This case report exemplifies the successful use of this therapy on a patient with postoperative chronic neuropathic facial pain. However, additional studies are needed to determine the role of carboxytherapy in pain management.
